# Estimating length of stay in publicly-funded residential and nursing care homes: a retrospective analysis using linked administrative data sets

**DOI:** 10.1186/1472-6963-12-377

**Published:** 2012-10-31

**Authors:** Adam Steventon, Adam Roberts

**Affiliations:** 1 The Nuffield Trust, 59 New Cavendish Street, London, W1G 7LP, UK

**Keywords:** Care home, Length of stay, Administrative data, Long-term care

## Abstract

**Background:**

Information about how long people stay in care homes is needed to plan services, as length of stay is a determinant of future demand for care. As length of stay is proportional to cost, estimates are also needed to inform analysis of the long-term cost effectiveness of interventions aimed at preventing admissions to care homes. But estimates are rarely available due to the cost of repeatedly surveying individuals.

**Methods:**

We used administrative data from three local authorities in England to estimate the length of publicly-funded care homes stays beginning in 2005 and 2006. Stays were classified into nursing home, permanent residential and temporary residential. We aggregated successive placements in different care home providers and, by linking to health data, across periods in hospital.

**Results:**

The largest group of stays (38.9%) were those intended to be temporary, such as for rehabilitation, and typically lasted 4 weeks. For people admitted to permanent residential care, median length of stay was 17.9 months. Women stayed longer than men, while stays were shorter if preceded by other forms of social care. There was significant variation in length of stay between the three local authorities. The typical person admitted to a permanent residential care home will cost a local authority over £38,000, less payments due from individuals under the means test.

**Conclusions:**

These figures are not apparent from existing data sets. The large cost of care home placements suggests significant scope for preventive approaches. The administrative data revealed complexity in patterns of service use, which should be further explored as it may challenge the assumptions that are often made.

## Background

Many parts of the world are expecting increases in the number of people who require long-term social care support for activities such as bathing or dressing [1]. Admissions to care homes can be undesirable for individuals who wish to live independently in the community. They can also be expensive. In England, efforts have been made to prevent admissions to care homes, but over 170,000 older people are residents receiving publicly-funded support [2]. Care homes constitute around 52% of gross local authority spending on social care services for older people (£4.7 billion) [3].

Given the weekly cost of care homes (£500 a week or more depending on the type of home), [4] when planning services it is important to understand how long people stay resident. Length of stay determines turnover and therefore is a determinant of demand for care homes in subsequent years [5]. Further, interventions designed to prevent admission to care homes are often funded based on anticipated reductions in future costs, so their evaluation requires knowledge about how long those care home stays might last. Unfortunately, basic information on care home length of stay has not been available, as statutory returns are often produced on a cross-sectional basis, and few longitudinal surveys of care home residents are available. Simply surveying current residents about completed length of stay will under-represent shorter stays, so bias findings towards longer stays.

One survey repeatedly followed up 2,573 people who were admitted to a care home in 1995 and found a median length of stay 19.6 months [6]. However eligibility criteria for social care have changed substantially over the past 17 years [7]. Given the cost of surveying large numbers of individuals over time, administrative data offer a promising alternative. A recent study used administrative data from a large provider of care homes, relating to people discharged from a care home during 2008–2010 [8]. This found shorter median stay than the 1995 study, at 15.2 months. However, the method could not take account of portions of care home stays provided by other providers, so may understate length of stay. Previous studies in the United States warned of the need to aggregate across short breaks outside of care homes, as well as across multiple stays [9]. For example, in operational data sets, care home stays may be terminated when individuals are admitted to hospital, even if care home beds will need to be available on their return.

We develop a method using administrative data from local authorities, who pay for social care under the means-test that operates in England, rather than data from providers. Further, we use recent advances in data linkage to obtain person-level histories that span both social care and hospital care.

## Methods

### Study areas and cohorts

We studied social care paid by three local authorities in England: a seaside town with a population of 135,000, a rural area with a population of 750,000 and an outer London suburb with a population of 340,000. The first one has a relatively high use of care homes (residents per 1,000 people at 60 percentile nationally) while the final two have relatively low use (25 percentile). There has been a large shortfall in residential and nursing care places in London compared to the rest of the country [10].

The data sets covered different periods in each site (December 2005 to November 2008, April 2005 to March 2008, September 2005 to August 2008). We excluded people with a record of care home use in the first three months of the data to ensure that we identified new entrants, and estimated length of stay for people admitted during the following twelve months. Using a twelve month period reduced the scope for seasonal effects. We restricted the sample to people aged 65 or over.

In England, care home placements are categorised as “residential” or “nursing”, with the latter including nursing support for medical needs. Some residential placements are categorised as “temporary”, which may include respite care, rehabilitation, short breaks and other care which is intended to be of a temporary nature [11]. Other placements are categorised as “permanent”. The categorisation is based on the intended nature of the admission at the outset, rather than on how long people ultimately stay. We distinguished between length of permanent residential, temporary residential and a single category of nursing placements; where an individual experienced a succession of placements, we categorised stays according to the nature of the first placement.

### Data sets

We used administrative data sets maintained by the local authorities. These were extracted from operational systems such as *Swift* and contain records of social care “service elements”, which relate to items of social care received for periods of time. No direct measure of social care need was consistent across the sites. Staff in the local authorities helped us identify records corresponding to residential and nursing care placements.

Social care data were linked to data on registrations with general practices sourced from local Primary Care Trusts (also known as ‘Exeter files’). This was done to restrict the sample to local residents (who were assumed to be registered with a local general practice) and to people aged 65 or over. General practice registration data also included date of death. We obtained data on inpatient, outpatient, and accident and emergency hospital use from the Secondary Uses Service.

All of the data sets were “pseudonymised” before transfer to the research team, in order to protect patient confidentiality. Thus, identifiable fields (such as name and address) were removed and an individual identifier was encrypted using a hash algorithm. In two of the sites, a national unique patient identifier (the “NHS number”) was available on both health and social care data sets and this was used as the basis for the data linkage. For the London suburb, we used an alternative identifier based on initials, sex, and date of birth.

These data sets were originally collected to test the feasibility of building a predictive model for social care [12]. The National Information Governance Board confirmed that individual consent was not required to link these data for the current study. The local authorities and Primary Care Trusts agreed to the reuse of the data. Ethical approval was not required as this was a retrospective study aimed at informing future policy and research methods.

### Analysis

Person-level files were created to show individuals’ pathways across health and social care and in particular admissions and discharges from care homes. We aggregated care home stays in multiple providers and stays that were interrupted by a hospital admission or a brief period outside of institutional care (lasting 30 days or less). Where a date of death was recorded in the general practice registration data, care home stays that were not already closed in the local authority system by this date were terminated.

Social care data contained all services received at any point within a three year window. We set aside the last month of the data set to ensure that discharges from care homes were not followed by a re-admission within 30 days. Thus, we were able to observe stays for between 20 and 32 months, depending on the date of admission. Longer stays were “censored” at the end of the data set, meaning that that these observations provided only partial information about length of stay. Kaplan-Meier curves were used to estimate length of stay allowing for censoring [13].

The relationship between individual characteristics and length of stay was analysed using multivariate Cox regression, [14] with the instantaneous rate (“hazard”) of discharge from care homes taken to be the dependent variable. Independent variables included age at admission, sex, an area-based socioeconomic deprivation score (national quartiles of the Index of Multiple Deprivation 2004 [15]), as well as social care and secondary care use during the three months before care home admission. The area-based deprivation score was assigned based on the characteristics of patients registered at the general practice. Compared to using a score based on the address of the care home, this was expected to reflect better the characteristics of individuals before admission. The regression pooled data across all three sites and we tested formally for differences between areas using the likelihood ratio.

Care home lengths of stay will tend to be correlated for individuals within the same local authority area. This could be due, for example, to intrinsic differences in policy frameworks or care home supply. To allow for these correlations, the regressions were done in a multilevel modelling framework. Specifically, the Cox regression included random effects (frailties) at the site level, which were assumed to be gamma distributed [16] to allow for correlation of length of stays within site.

### Costing

We estimated the cost of care home placements by applying daily unit costs, allowing for changes between residential and nursing home categories over time. Unit costs were assumed to be £497 per week for permanent residential care homes and £719 per week for nursing care homes, based on fees charged in privately-provided homes, before payments made by individuals under the means-test [4]. National average unit costs were applied in each site to allow robust comparison of the magnitude of care between areas; costs in London may be 12-19% larger than nationally [4]. Unit costs for temporary residential care were assumed to be the same as for permanent residential care.

Care home stays were sometimes interrupted by hospital stays or brief periods outside institutional care (lasting 30 days or less). Two scenarios are presented for care home costs while outside of the care home. The first assumes that costs continue to accrue at the same rate as before; this corresponds to a bed being reserved for the individual on their return. The second assumes nil costs during the period outside of the care home; the bed is occupied with another individual or the local authority does not incur costs due to empty beds. Median costs were estimated using Kaplan-Meier curves [13].

## Results

Across the three sites, we identified 3,421 care home admissions within the twelve-month periods selected. The biggest category was temporary residential care placements, constituting 38.9% of the sample. Permanent residential care placements made up 36.4% of the sample, with the remaining 24.7% being nursing home placements.

Tables 1 and 2 show the characteristics of people admitted. Age at admission was similar between permanent residential and nursing homes on average (85.8 years compared with 85.0), though people admitted to permanent residential homes were more likely to be female (72.4% compared with 62.6%). Permanent admissions to residential care were often preceded by domiciliary care or emergency hospital admission (37.6% and 45.1% of people, respectively). In comparison, people admitted to nursing homes were less likely to have received domiciliary care (26.2%) but more likely to have had an emergency hospital admission (56.7%). Mean socioeconomic deprivation scores were similar across all categories (19.8, 19.3 and 18.8 for temporary residential care, permanent residential care and nursing care, respectively).

**Table 1:** **Characteristics of people admitted to temporary and permanent residential care homes** (**data are percentages unless otherwise stated**; **SD** = **standard deviation**) (**N**=**2**,**576**)

	**Temp.**	**Temp.**	**Temp.**	**Temp.**	**Perm.**	**Perm.**	**Perm.**	**Perm.**
	**Coastal town** (**n=380**)	**Rural county** (**n=820**)	**London suburb** (**n=131**)	**All sites** (**n=1,331**)	**Coastal town** (**n=198**)	**Rural county** (**n=880**)	**London suburb** (**n=167**)	**All sites** (**n=1,245**)
Mean age in years (SD)	85.1 (7.5)	84.1 (6.9)	83.5 (20.3)	84.3 (7.2)	85.9 (7.9)	86.4 (17.7)	83.1 (7.6)	85.8 (7.2)
65-79	20.5	22.4	31.3	22.8	21.7	13.6	34.7	17.8
80-84	23.2	28.8	20.6	26.4	13.1	23.8	20.4	21.6
85-89	26.3	25.2	25.2	25.5	28.8	27.8	24.6	27.6
90+	30.0	23.5	22.9	25.3	36.4	34.8	20.4	33.1
Female	66.1	75.4	66.4	71.8	66.2	74.3	70.1	72.4
Mean deprivation (IMD 2004) score (SD)*	26.0 (3.5)	16.8 (4.0)	20.3 (7.5)	19.8 (5.9)	26.1 (3.5)	17.7 (4.2)	20.0 (7.9)	19.3 (5.7)
1st quartile	0.0	0.4	4.6	0.7	0.0	0.0	1.8	0.2
2nd quartile	1.6	54.1	32.8	37.0	1.5	48.1	41.9	39.8
3rd quartile	84.2	45.2	51.9	57.0	80.3	51.1	43.1	54.7
4th quartile	14.2	0.2	10.7	5.3	18.2	0.8	13.2	5.2
Service use in 3 months before admission								
Day care	12.4	2.4	3.8	5.4	13.1	16.9	4.2	14.6
Domiciliary care	24.7	15.7	6.9	17.4	39.4	40.9	18.0	37.6
Other social care	12.1	11.0	6.9	10.9	24.7	20.5	28.7	22.2
Emergency admission	22.1	62.9	31.3	48.2	37.4	47.0	43.7	45.1
Elective admission	6.1	18.8	11.5	14.4	6.1	13.0	4.8	10.8
Outpatient attendance	26.1	44.1	32.1	37.8	24.2	27.8	34.1	28.1

* Based on Index of Multiple Deprivation 2004 of the general practice. First quartile is least deprived; fourth quartile is most deprived.

**Table 2:** **Characteristics of people admitted to nursing care homes** (**data are percentages unless otherwise stated**; **SD** = **standard deviation**) (**N**=**845**)

	**Coastal town** (**n**=**103**)	**Rural county** (**n**=**573**)	**London suburb** (**n**=**169**)	**All** (**n**=**845**)
Mean age in years (SD)	86.3 (6.9)	84.6 (7.1)	85.5 (8.5)	85.0 (7.4)
65-79	16.5	20.8	24.3	20.9
80-84	25.2	27.9	20.1	26.0
85-89	19.4	25.8	17.8	23.4
90+	38.8	25.5	37.9	29.6
Female	70.9	59.0	69.8	62.6
Mean deprivation (IMD 2004) score (SD)*	25.8 (3.6)	17.2 (4.1)	20.2 (6.7)	18.8 (5.5)
1st quartile	0.0	0.2	4.1	0.9
2nd quartile	2.9	47.6	30.8	38.8
3rd quartile	77.7	51.8	56.2	55.9
4th quartile	19.4	0.3	8.9	4.4
Service use in three months before admission				
Day care	6.8	12.4	1.2	9.5
Domiciliary care	30.1	29.7	11.8	26.2
Other social care	14.6	13.4	10.7	13.0
Emergency admission	37.9	57.8	64.5	56.7
Elective admission	6.8	16.1	3.6	12.4
Outpatient attendance	24.3	29.7	23.7	27.8

* Based on Index of Multiple Deprivation 2004 of the general practice. First quartile is least deprived; fourth quartile is most deprived.

We observed a total of 1.0 million person-days in care homes. Across all sites, 2,755 stays (80.5%) ended within the period covered by the data set, with the remaining censored at the end of the period. A significant proportion (42.5%) of temporary residential care stays were followed by a community-based social care service (for example, domiciliary/home care or day care). Over half of permanent and nursing home stays ended in the person dying in the home or within 30 days (50.3% and 54.8%, respectively) (Table 3). Some stays were not followed by an indication that the person had died or moved on to receive some other form of social care service. These may have transferred to care homes that were paid for privately or by another local authority. Alternatively, mortality data from the general practice registration may have been incomplete.

**Table 3:** **Destination after discharge from care homes for completed care home stays** (**data are** % **of stays**)

	**Community**-**based social care within 30 days**	**Death within 30 days**	**Other**
**Temporary residential care**			
All sites (n=1314)	42.5	6.0	51.4
Coastal town (n=378)	42.3	11.1	46.6
Rural county (n=805)	43.0	3.0	54.0
London suburb (n=131)	40.5	9.9	49.6
**Permanent residential care**			
All sites (n=781)	4.4	50.3	45.3
Coastal town (n=138)	6.5	63.0	30.4
Rural county (n=553)	2.7	49.0	48.3
London suburb (n=90)	11.1	38.9	50.0
**Nursing care**			
All sites (n=660)	12.0	54.8	33.2
Coastal town (n=79)	13.9	50.6	35.4
Rural county (n=468)	12.6	53.0	34.4
London suburb (n=113)	8.0	65.5	26.5

**Figure 1:**
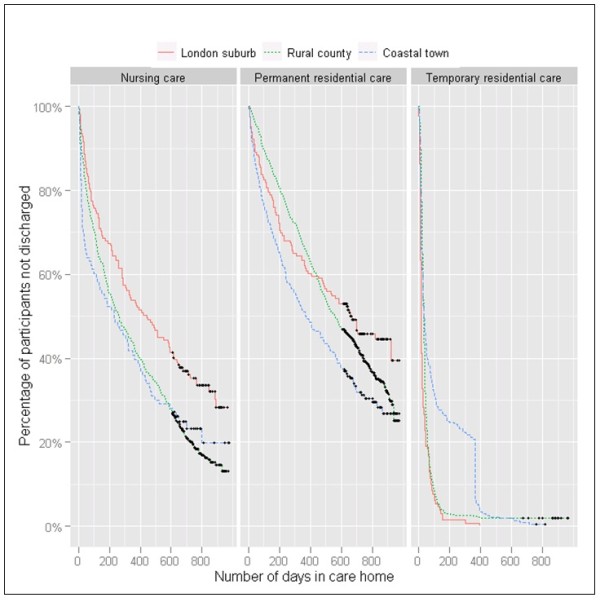
Kaplan-Meier curves.

Figure 1 shows Kaplan-Meier curves for length of stay. Median length of stay in temporary residential care was 15 days in the London suburb and around one month (34.5 and 32.0 days) for the other sites (Table 4). Median permanent residential length of stay was longer than median nursing home length of stay in every site.

**Table 4:** **Kaplan**-**Meier estimates of length of stay**

	**25 percentile**	**Median**	**75 percentile**
**Temporary residential care**			
All sites	16.0 (14, 17)	31.0 (28, 33)	59.0 (53, 67)
Coastal town	14.0 (undefined)	34.5 (28, 42)	192.5 (112, 364)
Rural county	20.0 (18, 20)	32.0 (30, 34)	48.0 (45, 53)
London suburb	8.0 (7, 11)	15.0 (13, 18)	39.0 (24, 59)
**Permanent residential care**			
All sites	219.0 (193, 241)	544.5 (495, 586)	*
Coastal town	116.0 (71, 161)	369.0 (271, 516)	*
Rural county	254.5 (230, 294)	559.0 (503, 617)	*
London suburb	170.0 (113, 230)	671.0 (483, undefined)	*
**Nursing care**			
All sites	73.0 (57, 90)	283.0 (243, 330)	693.0 (636, 766)
Coastal town	22.0 (14, 42)	231.0 (118, 363)	649.0 (458, undefined)
Rural county	73.0 (57, 95)	261.0 (218, 315)	636.0 (586, 693)
London suburb	118.0 (65, 174)	440.0 (290, 585)	*

*Could not be estimated as fewer than 75% of patients were discharged within the period covered by the data set. Data are in days (95% confidence interval).

Cox regression confirmed that nursing home stays were shorter than permanent residential stays after controlling for individual characteristics (Table 5). Women had longer stays, while prior domiciliary care and prior elective (i.e. planned) hospital admissions were associated with shorter stays. Only one deprivation quartile (3rd) was associated with statistically-significant length of stay than the least deprived reference category. Further, coefficients did not show any clear trend across quartiles. The likelihood ratio test revealed statistically-significant differences by site (theta=0.0052, p=0.021).

**Table 5:** **Cox regression** (**hazard ratio is for the hazard of leaving care home**)

	**Hazard ratio**	**95%confidence interval**	**p**
Temporary residential*	7.78	7.00 to 8.65	0.000
Nursing*	1.61	1.45 to 1.79	0.000
Age 80–84 years**	0.96	0.85 to 1.07	0.440
Age 85–89 years**	1.01	0.90 to 1.13	0.914
Age 90+ years**	1.11	0.99 to 1.24	0.063
Deprivation 2nd quartile***	0.67	0.42 to 1.05	0.078
Deprivation 3rd quartile***	0.57	0.36 to 0.89	0.014
Deprivation 4th quartile***	0.69	0.43 to 1.11	0.128
Female****	0.85	0.78 to 0.92	0.000
Prior day care	1.03	0.90 to 1.17	0.696
Prior domiciliary care	1.10	1.01 to 1.21	0.033
Other prior social care	0.96	0.86 to 1.07	0.498
Prior emergency admission	1.06	0.98 to 1.15	0.127
Prior elective admission	1.33	1.19 to 1.49	0.000
Prior oupatient attendance	1.01	0.93 to 1.09	0.893

* Hazard ratios are relative to permanent care home admissions as the reference category.

** Hazard ratios are relative to age 65–79 years as the reference category.

*** First quartile is least deprived; fourth quartile is most deprived. Hazard ratios are relative to the first quartile as the reference category.

**** Hazard ratio is relative to males.

The costing algorithm could be applied to 3,400 out of 3,421 admissions (99.4%). Median costs were £2,201 for temporary residential care stays, £38,624 for permanent residential stays and £29,479 for nursing home stays, when costs were assumed to continue to accrue during breaks from the home. When costs were assumed not to accrue, these figures were slightly lower (£1,917, £38,127 and £29,171, respectively).

## Discussion

This study has addressed the lack of information about care home length of stay by using administrative data. We found that residents often spent several years in care homes, with a median length of stay of 544.5 days (17.9 months) for permanent residential care. The biggest category of stays, however, was temporary placements, which constituted 38.9% of the total. These typically lasted 4 weeks, though 25% lasted longer than 8 weeks.

Compared to residential stays, nursing home stays were more likely preceded by emergency hospital admission (56.7% compared with 45.1%), perhaps reflecting the development of care needs requiring medical care in a care home. Stays in care homes tended to be shorter for people who had previously received domiciliary social care, although whether this was because of successful preventive care delaying admission or higher levels of need (and shorter life expectancy) was not possible to say. Women tended to have longer stays than men, perhaps because they are less likely to have a spouse at older ages [17].

The median length of stay observed for our sample of permanent residential home placements (17.9 months, 95% CI, 16.2 to 19.3 months) was significantly shorter than that reported in the 1995 longitudinal study (26.8 months) [6]. The same was true for nursing homes, though differences were smaller (9.3 months, 95% CI, 8.0 to 10.8, compared with 11.9 months). As our study was restricted to publicly-funded care, it does not reflect total length of stay as people may transfer between privately-funded and publicly-funded care (for example, when assets are depleted to the extent that a person qualifies for means-tested public support). Further, people may transfer to care funded by a different local authority. Differences between our estimates and the older 1995 survey might also reflect changes over time in eligibility criteria for public support. Thus it is possible that people are now admitted to care homes later in life, so remain in the care home for shorter periods of time. This was borne out by a comparison of average age at admission (85.8 for residential placements and 85.0 for nursing placements in our sample, compared with 83.5 and 82.5 in the 1995 study, respectively).

Compared to previous studies, the method used in the current study more clearly highlights differences in length of stay between geographic areas. These were statistically significant after adjusting for the observed individual characteristics. Compared with the other areas, a smaller proportion of care home stays in the London suburb were temporary (28.1% compared with 55.8% and 36.1%) and the median length of temporary stays was shorter (15 days compared with around a month). One explanation is that policy might be less heavily focused on the use of short-term care in the London suburb than the other areas, perhaps because of the lower levels of care home supply [10]. This might explain why permanent residential care stays were typically longer in this site than in the others, if the available beds are targeted on those who need support for longer. Alternatively, the combination of long stayers in permanent residential care and the limited supply might have constrained the local care system so that it was only able to offer temporary stays to a minority of clients. As no consistent measure of social care need was available from the administrative data, a further study would be needed to test these hypotheses.

Another advantage of the method is that it aggregates over successive care home placements. Compared with cross-sectional methods, we were better able to reflect the preponderance of short stays, which formed the biggest single category of stays. As we focus on cohorts of admissions rather than discharges, we reflect relatively recent policies about eligibility criteria.

The administrative data allowed for longitudinal analysis across different elements of service use, but the quality of these data were not under our control. For example, one of the Kaplan-Meier curves showed a distinct drop corresponding to 365 days, which is likely to be an artefact of the data. Further efforts might be made to increase recording of standardised measures of social care need in particular, as this will increase the usefulness of these data. Comparisons of self-report and administrative data on social care use might also be undertaken, as has happened for health services [18].

The findings illustrate the complexity of patterns of service use. For example, discharges from temporary residential care placements were often made to community-based services. For permanent residential and nursing stays, we found a significant proportion of stays were not followed by an identified death or other service. This appears to contradict assumptions often made that people remain residents until death [19]. However, there may be incomplete recording of deaths on primary care data. Efforts have been made to improve recording [20] but these have not been specifically targeted on former residents of care homes as far as we are aware. It is also possible that residents transferred to care funded privately or by another local authority. The latter would mean length of stay of publicly supported residents was understated.

The study has demonstrated one potential use of linked administrative data sets. However, the limited availability of these data sets for research meant that we were limited to three local authority areas. Although these areas included a mix of settings and rates of care home use, they are unlikely to be representative of the country as a whole. Further, it was not possible to track care home residents who began to be funded by another local authority. Therefore, the availability of nationally-collated standardised data at the person level would be beneficial.

Our findings suggest that a person admitted to a permanent care home will cost a local authority over £38,000 on average, less means-tested user payments. This figure would not be apparent from available data on service use, which is often cross-sectional rather than across the lifetime. Our findings suggest that substantial effort may be warranted into developing preventive interventions, as the cost of providing care homes is very high. However, the evaluation of interventions must take into account outcomes for individuals as well as cost and compare different alternatives. We note that the evidence for effective prevention of admission into residential and nursing care homes is often weak, [21] though models exist to target interventions on those most likely to be admitted in the absence of additional support [12].

## Conclusions

Permanent residential placements typically lasted 18 months. Therefore, a substantial proportion of current residents of care homes will continue to require support in future years. Robust data about length of stay have rarely been available, so our findings should be taken into account when planning services and in models developed for estimating future demand for services [22]. They should also be taken into account in cost-effectiveness studies aimed at establishing the long-term impacts of interventions aimed at preventing admissions to care homes.

The longitudinal data revealed substantial variations in patterns of service use between areas. These will have implications for Government policy about funding of care, for example for the feasibility of setting lifetime caps on care costs that do not vary between areas [23]. However further studies are needed to determine the factors associated with variations in the use of services. Administrative data may have a role to play and its usefulness would be increased with more consistent data on social care needs.

## Competing interests

The authors declare that they have no competing interests.

## Authors' contributions

AS designed the study, conducted statistical analyses and drafted the manuscript. AR coded and structured the administrative data, had input into the analyses and helped to draft the manuscript. Both authors read and approved the final manuscript.

## Pre-publication history

The pre-publication history for this paper can be accessed here:

http://www.biomedcentral.com/1472-6963/12/377/prepub
